# Effect of Bariatric Surgery on Glycaemic Control in King Fahad Hospital

**DOI:** 10.7759/cureus.18943

**Published:** 2021-10-21

**Authors:** Mohammed Khaled S Zaki, Rayan E Alhelali, Yazeed H Samman, Abdulaziz Saud Alharbi, Yazeed K Alharbi, Abdullah K Alrefaei, Mohammed I Hasaballah, Osama A Alquliti

**Affiliations:** 1 General Surgery, Taibah University, Al-Madinah Al-Munawwarah, SAU; 2 College of Medicine, Taibah University, Al-Madinah Al-Munawwarah, SAU

**Keywords:** glycaemic control, type 2 diabetes mellitus, obesity, bariatric surgery, laparoscopic sleeve gastrectomy

## Abstract

Background: Laparoscopic Sleeve Gastrectomy (LSG) is an approved procedure for weight reduction in obese patients. This outcome of weight loss is essential to achieve optimal control in patients with type 2 diabetes mellitus (T2DM).

Objectives: This study was designed to evaluate the effect of LSG on glycemic control among a sample of obese patients in Al-Madinah Al-Munawwarah, Saudi Arabia, through assessment of reduction in hemoglobin A1c (HbA1c) associated with weight loss following LSG.

Methods: In this cross-sectional study, we studied 102 patients with a body mass index (BMI) of ≥30 kg/m^2 ^and aged ≥18 years who underwent LSG between January 2017 and December 2019. Patient age, characteristics, preoperative and postoperative records of BMI and HbA1c were collected. The data of BMI and HbA1c were analyzed based on baseline and mean postoperative readings with variable postoperative visits after LSG.

Results: There was a 30% reduction in BMI and a 26.4% reduction in HbA1c following LSG from baseline in all patients. We noted 44 patients achieved BMI <40kg/m^2^ with HbA1c <6.5% and 32 patients achieved BMI <40kg/m^2^ with HbA1c <5.7% within a mean follow-up time of 10 months.

Conclusions: Laparoscopic Sleeve Gastrectomy (LSG) has a positive effect on glycemic control in obese patients in short term, evidenced by the significant reduction of weight and HbA1c. Larger longitudinal studies are needed to assess the long-term impact of LSG glycemic control and the related factors associated with maintaining weight reduction and optimal glycemic control in Saudi Arabia for patients with obesity.

## Introduction

One of the most significant systemic diseases with a devastating impact on quality of life around the globe is obesity [1]. The prevalence of obesity in the Kingdom of Saudi Arabia (KSA) climbs across all age groups over 15 years old with predominance for females [2]. Multiple factors are identified in developing obesity including diet as a major factor, followed by lack of physical activity and marital status [3]. A variety of co-morbidities are associated with obesity, including type 2 diabetes mellitus (T2DM) [4]. Saudi Arabia is listed by WHO as the seventh country worldwide and the second in the Middle East to have high rates of diabetes with more than six million patients plus more than two million with pre-diabetes [5]. Most of the diabetic patients in Saudi Arabia suffer from obesity [2].

In an effort to handle the burden of obesity, the Saudi government has dedicated 500 million riyals in 2018 for obesity prevention and management program [6]. As physical activity is a contributing factor to obesity, it was found that physical inactivity is high among Saudi females, due to several cultural factors prohibiting exercise and practicing sports in public [7]. Significant advances in diabetes management contributed to achieving control, whereas some cases still remain difficult to reach the goal [8]. Weight loss has been described as an effective method of controlling and/or preventing diabetes [9]. There are many pharmacological and nonpharmacological strategies being used in the management and prevention of obesity. However, previous literature reported that some patients failed to sustain weight loss in the long term [10]. 

Bariatric surgery is a recognized intervention for weight loss with a reduction of co-morbidities in patients with obesity [11]. Several international studies revealed the positive outcomes of bariatric surgery [12]. These studies outline the significant reduction of hemoglobin A1c (HbA1c), serum glucose, and cardiovascular risk in diabetic patients [13-16]. In addition, some studies revealed remission of diabetes in obese patients who underwent bariatric surgery along with medical therapy and lifestyle modifications compared to medical therapy alone [17]. There are several types of bariatric surgery which are malabsorptive procedures, restrictive procedures, or a combination of these two types, and all these types lead to weight loss through changes in metabolism with different mechanisms [18]. Laparoscopic Sleeve Gastrectomy (LSG) is a worldwide acknowledged surgical modality to manage obesity with a positive outcome in diabetic patients [19]. This procedure is a restrictive surgery to reduce weight by changing the physiology of appetite and gastric emptying [20]. Moreover, LSG was demonstrated to have a short operative time with a low risk of complications [21]. This modality of treatment should be a part of a comprehensive program of weight reduction, with long-term medical monitoring and lifestyle support [22]. In Saudi Arabia, there is a paucity of studies that document the outcome of bariatric surgeries on weight reduction and HbA1c. Ahmed et al. (2018), concluded that there is a substantial reduction in HbA1c between preoperative and postoperative periods within 12 months among obese patients who underwent LSG, and it was explained by the achieved reduction of BMI [23]. Because of its effectiveness in weight loss, LSG became the preferable procedure in Saudi Arabia.

The aim of this research is to evaluate the effect of LSG on glycemic control among a sample of obese patients in Al-Madinah Al-Munawwarah, Saudi Arabia, through assessment of reduction in HbA1c associated with weight loss following LSG.

## Materials and methods

This cross-sectional research was conducted following the approval of the Institutional Review Board at King Fahad Hospital Research, Al-Madinah Al-Munawwarah, Saudi Arabia, the Institutional Review Board at General Directorate of Health Affairs in Al-Madinah Al-Munawwarah, Saudi Arabia (IRB 534), and the Scientific Research Ethics Committee at Taibah University, Saudi Arabia (PEP4-M6-1441). In this research, 102 patients were included according to the following inclusion criteria: underwent LSG in the period between January 2017 and December 2019 with BMI ≥30 kg/m^2 ^and HbA1c ≥ 5.7% before the surgery; the patient underwent the surgery at King Fahad Hospital in Al-Madinah Al-Munawwarah, Saudi Arabia; and the LSG procedure was offered to all patients electively and was followed postoperatively with variable follow-up periods.

The indication for surgery for these patients was according to Saudi guidelines on the prevention and management of obesity. Regarding indications for bariatric surgery, the procedure is indicated for patients with BMI more than 40, or 35 kg/m^2^ with obesity-related co-morbidities such as hypertension, T2DM, obstructive sleep apnea, and for those who fail to improve with non-surgical treatment [24].

BMI of all patients was calculated for the purpose of identification, categorization, and analysis of the data. The data of all patients were retrieved from their existing medical records. Informed consent was not needed, and all data were kept confidential and secured with no identifier. The characteristics of the patients and the baseline data were collected including age, gender, diabetic state, height, weight, and HbA1c. The most recent record of HbA1c and the calculated BMI before the procedure were considered as a baseline. Any patient who underwent LSG during the period of the study with missed data of height, weight, and/or HbA1c was excluded.

The authors divided the patients into two groups: diabetic patients and prediabetic patients, and they further categorized them according to their baseline BMI before LSG into patients with BMI ≥40 kg/m^2^ and patients with BMI <40kg/m^2^. The prediabetic patients in this study were identified as those with baseline HbA1c levels between ≥5.7% and <6.5%. The effect of LSG was evaluated using (baseline - mean postoperative records) of BMI and HbA1c according to the available data for each patient irrespective of the follow-up period. Not all patients had complete follow-up records of one year, which forced the authors to calculate a mean follow-up period for all patients. The mean follow-up period was 10 months. The authors used the criteria of the American Association of Diabetes as HbA1c <6.5% in diabetic patients and HbA1c <5.7% in prediabetic patients for achieving normal glucose levels.

Data analysis was performed using the statistical package for social sciences (SPSS) software, version 21 (IBM Corp., Armonk, NY). Normality tests were conducted using the Shapiro-Wilk test. The data followed an abnormal distribution, so, a non-parametric test was applied. Qualitative data were presented using counts and percentages, while quantitative data were presented using mean ± Standard Deviation (SD). Paired T-test was used to compare preoperative and postoperative data of BMI and HbA1c in diabetic and prediabetic groups. As well as comparing the preoperative and postoperative data of BMI and HbA1c in patients with BMI ≥40kg/m^2^ versus patients with BMI <40kg/m^2^ before LSG. A p-value of ≤0.05 (two-sided) was used to indicate statistical significance.

## Results

This study included 102 obese patients who underwent LSG during the period of the study and fulfilled the inclusion criteria. The characteristics information of the patients at baseline are detailed in Table 1. The mean age was 41.6±12.2 with a range of 19 to 64 years old, and most of the patients were females (72.5%). In our sampled patients, 64.7% had diabetes and 35.3% were prediabetic. The majority of the patients had a baseline BMI ≥40kg/m^2^ (80.4%). The mean baseline of BMI was 47.3±7.73 kg/m^2^, and the range of HbA1c was between 5.7% and 12.2% at baseline.

**Table 1:** Baseline characteristics of patients (n=102) N = Number of patients; n = Total number of patients

Study variables	N (%)
Gender	
Male	28 (27.5%)
Female	74 (72.5%)
Diabetes	
Yes	66 (64.7%)
No	36 (35.3%)
BMI ≥ 40kg/m^2^	
Diabetic	50 (49%)
Non-diabetic	32 (31.4%)
BMI < 40kg/m^2^	
Diabetic	16 (15.7%)
Non-diabetic	4 (3.9%)
	Mean ± SD
Age (19 – 64) years	41.6 ± 12.2
Baseline BMI (30.9 – 67.4)kg/m^2^	47.3 ± 7.73
Baseline HbA1c (5.7 – 12.2)%	7.45 ± 1.66

The paired T-test comparison between baseline and postoperative BMI and HbA1c among all patients is shown in Table 2. We found that there was a statistically significant difference between preoperative and postoperative BMI (mean differences: 14.2; p<0.001). Also, a significant difference was found in the comparison between preoperative and postoperative HbA1c (mean differences: 1.67; p<0.001).

**Table 2:** Paired T-test of Pre- and Postoperative BMI and HbA1c of all patients (n=102) ** = Statistically significant; n = Total number of patients

Variables	Baseline Mean ± SD	Postoperative Mean ± SD	Mean Differences	P-value
BMI (kg/m^2^)	47.3 ± 7.73	33.1 ± 6.72	14.2	<0.001 **
HbA1c (%)	7.45 ±1.66	5.78 ± 0.92	1.67	<0.001 **

**Figure 1:**
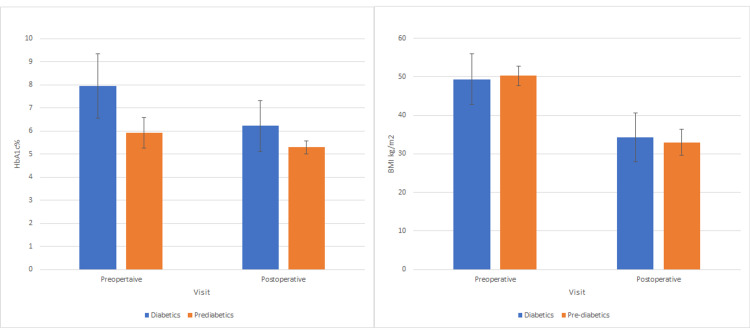
Baseline and postoperative means of HbA1c and BMI in patients with BMI ≥40kg/square meters before LSG HbA1c = Hemoglobin A1c; BMI = Body mass index; LSG = Laparoscopic sleeve gastrectomy

The number of patients with BMI ≥40 kg/m^2^ before LSG was 82 and with BMI <40 kg/m^2 ^was 20. Diabetic patients with BMI ≥40 kg/m^2^ were 50, while 32 were prediabetic. Moreover, there were 16 diabetic patients with BMI <40 kg/m^2^ and four patients were prediabetic. Baseline and postoperative means of HbA1c and BMI in patients with BMI ≥40kg/m^2^ before LSG are presented in Figure 1. In the paired T-test of this group, there is a significant difference between preoperative and postoperative values for BMI and HbA1c in both diabetic and prediabetic patients, with a higher decrease in HbA1c in the diabetic group compared to the prediabetic group (mean differences 1.72 vs. 0.63, p=0.001 vs. p=0.001), and a higher weight loss in the prediabetic group compared to the diabetic group (mean differences: 17.30 vs. 15.10, p=0.001 vs. p=0.001).

**Figure 2:**
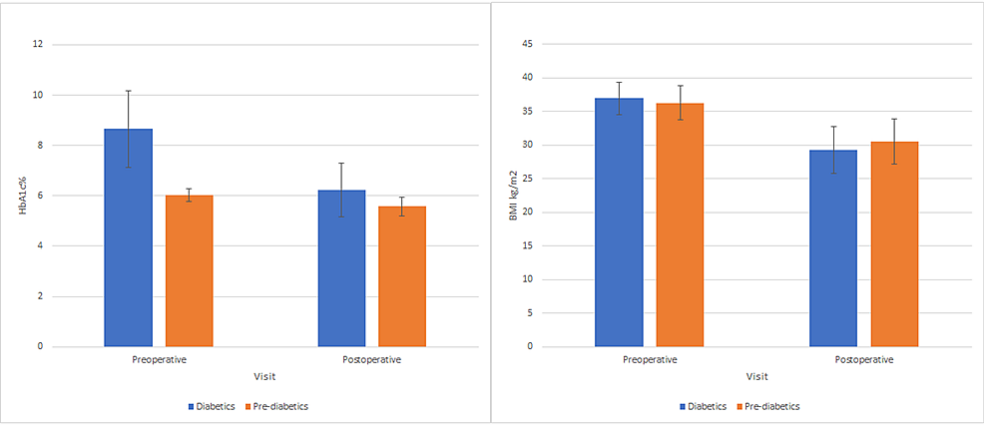
Baseline and postoperative means of HbA1c and BMI in patients with BMI <40kg/square meters before LSG HbA1c = Hemoglobin A1c; BMI = Body mass index; LSG = Laparoscopic sleeve gastrectomy

Data of baseline and postoperative means of HbA1c and BMI in patients with BMI<40kg/m^2^ are presented in Figure 2. There is a significant difference between preoperative and postoperative values of BMI in both diabetic and prediabetic groups, with higher weight reduction observed in the diabetic group compared to the prediabetic group (mean differences: 7.67 vs. 5.77, p=0.001 vs. p=0.027). There was a significant difference found between the preoperative and postoperative values of HbA1c in the diabetic group (mean difference: 2.44, p=0.001) but there was a non-significant difference in the prediabetic group.

## Discussion

In Saudi Arabia, there is a paucity of studies documenting the effects of bariatric surgeries on weight reduction and HbA1c. This cross-sectional study aimed to evaluate the impact of LSG on glycemic control among a sample of obese patients in Al-Madinah Al-Munawwarah, Saudi Arabia. Data of 102 patients with BMI ≥30kg/m^2^ who underwent LSG at King Fahad Hospital in Al-Madinah Al-Munawwarah, Saudi Arabia, were collected between January 2017 and December 2019. Weight loss procedures were generally reported to have a substantial effect on HbA1c in the short term (identified as a period of 12 months postoperatively).

In this study, LSG outcome showed improvement in both HbA1c and BMI postoperatively within a mean follow-up of 10 months. Following LSG, the number of patients with BMI <40kg/m^2^ was 86 (84.3%) in both diabetic and prediabetic groups. There was a 30% reduction in the BMI after LSG in all patients. Weight reduction was observed in 28.3% diabetic and 32.45% prediabetic patients. The reduction in HbA1c following the procedure was 26.4% for all patients (27.4% for diabetic and 11% for prediabetic patients).

As per the American Association of Diabetes criteria, diabetic patients with postoperative HbA1c <6.5% and prediabetic patients with postoperative HbA1c <5.7 % achieved target glycemic control. There were 76 patients (74.5%) who met the control level of HbA1c (<6.5%) after LSG. Also, there were 46 patients (45.1%) who reached the normal level of HbA1c (<5.7%) postoperatively. Depending on HbA1c, the total number of diabetic patients who achieved BMI <40kg/m^2^ with HbA1c <6.5% after LSG within the follow-up period was 44 (66.7%). For the prediabetic group, 32 patients (88.89%) reached a BMI <40kg/m^2^ with HbA1c <5.7% after LSG among those with BMI ≥40kg/m^2 ^preoperatively. As a result, LSG appears to be a preventive procedure in prediabetic patients with BMI ≥40kg/m^2^, but with a lack of enough evidence to confirm such effect. Among diabetic patients with BMI <40kg/m^2^ before LSG, 14 patients reached HbA1c <6.5% after surgery.

Previous literature reported a substantial reduction of BMI associated with HbA1c reduction within 12 months, and we noted similar results, but within 10 months [23,25,26]. Lee et al. (2009) found remission of diabetes in poorly controlled patients with a 22.8% reduction in HbA1c after LSG. This effect is related to decreased insulin resistance due to calorie restriction [27]. A study done by Ahmed et al. (2018) reported that patients with BMI >40 kg/m^2^ had a significant reduction in weight within 12 months after LSG, with a 25% reduction of weight postoperatively [23]. Also, Ahmed et al. (2018) and Sucandy et al. (2013) reported a significant postoperative reduction of BMI and improved glycemic control at one year following LSG [25,26].

Furthermore, Attia R (2019) reported that LSG has a positive outcome in the management of T2DM in patients with BMI < 35 kg/m^2^ and in those with BMI ≥ 35kg/m^2^ [22]. Thus, the findings of our study analysis are consistent with the previous research.

This literature studied the outcome of LSG concerning the regulation of insulin and fat metabolism. LSG involves fundus resection by longitudinal partial gastrectomy of about two-thirds of the stomach, reducing ghrelin secretion and appetite [20]. Through this mechanism, the fasting effect decreases insulin resistance resulting in improved serum glucose levels and fat metabolism [27]. Moreover, previous literature reported that the LSG procedure impacts the physiology of gastric emptying by accelerating the process, which contributes to weight loss [28].

As we noted the effect of LSG on the diabetic and prediabetic patients, the procedure showed a good effect on postoperative BMI and HbA1c in patients with BMI ≥ 40 kg/m^2^ before surgery and in diabetic patients with BMI < 40 kg/m^2^. Therefore, this procedure appears to be effective for glycemic control in diabetic patients and weight reduction in prediabetic patients in the short term. LSG was studied before and proved to have a low complication rate compared to other techniques with a positive effect on glycemic control and weight, making it a promising procedure in Saudi Arabia to manage diabetes in obese patients. The authors recommend further investigation of the effect of LSG on glycemic control in both diabetic and prediabetic patients and the related factors associated with maintaining weight reduction and optimal glycemic control in obese patients with diabetes.

Our study is limited by the study design, and the data were collected from a single center. Another limitation is that not all patients had complete follow-up data either because the patient had poor follow-up or the hospital had no documented data, so we attempted to analyze the data utilising the recorded data with a mean follow-up period. Also, any type of medications used by the patients and their possible interaction on BMI or HbA1c were not investigated. Moreover, we studied a small sample size of prediabetic patients, whereas a larger sample and more data would provide good insight into the outcome of LSG in this group.

## Conclusions

This study shows a positive effect of LSG on glycemic control in obese patients in short term, evidenced by the significant reduction in weight and HbA1c. The reduction in BMI leads to a reduction in HbA1c levels. Larger longitudinal studies should be conducted in Saudi Arabia regarding the long-term clinical outcomes of the surgical weight loss interventions, and the factors to sustain optimal weight and glycaemic control in obese patients. 
